# “*Living Through Two Storms*”: A Narrative Enquiry of Older Adults’ Experiences with HIV/AIDS During the COVID-19 Pandemic in Nigeria

**DOI:** 10.3390/jal5030023

**Published:** 2025-07-09

**Authors:** Olufisayo O. Elugbadebo, Oluwagbemiga Oyinlola, Baiba Berzins, Bibilola Oladeji, Lisa M. Kuhns, Babafemi O. Taiwo

**Affiliations:** 1 Department of Psychiatry, College of Medicine, University of Ibadan, Ibadan 200005, Nigeria; 2 School of Social Work, McGill University, Montreal, QC H3A 2T5, Canada; 3 Medical Social Services Department, University College Hospital, Ibadan 200005, Nigeria; 4 Division of Infectious Diseases, Northwestern University Feinberg School of Medicine Chicago, IL 60611, USA; 5 Department of Pediatrics, Feinberg School of Medicine & Ann & Robert H. Lurie Children’s Hospital of Chicago, Division of Adolescent and Young Adult Medicine, Northwestern University, Chicago, IL 60611, USA

**Keywords:** antiretroviral therapy (ART), older adults, HIV, COVID-19, lockdown

## Abstract

The COVID-19 pandemic has illuminated and intensified pre-existing structural vulnerabilities among older adults living with HIV/AIDS in sub-Saharan Africa, particularly Nigeria. Within already overstretched healthcare infrastructures, these individuals faced heightened economic precarity, disrupted HIV care, and pronounced psychosocial distress. Exploring their lived experiences critically advances an understanding of resilience and informs contextually responsive interventions that can mitigate future health crises. This study employed a narrative qualitative approach to explore the lived experiences of older adults (aged 50 and above) attending the Infectious Diseases Institute (IDI) clinic in Ibadan, Nigeria, during the pandemic lockdown. Purposive sampling guided by maximum variation principles enabled the selection of 26 participants who provided detailed accounts through in-depth interviews. Reflective thematic analysis identified complex narratives illustrating intensified financial hardships, disrupted access to antiretroviral therapy (ART), and heightened psychological distress, including anxiety, depression, and profound isolation. Conversely, participants also articulated experiences of resilience, manifesting in improved medication adherence, strengthened family bonds, and introspective growth fostered by enforced isolation. These nuanced findings highlights the necessity of developing an adaptive, integrated healthcare interventions that addresses economic vulnerabilities, psychosocial wellbeing, and ART continuity, thereby better preparing resource-constrained health systems to support older adults with HIV/AIDS in future public health crises.

## 1. Introduction

The advent of antiretroviral therapy (ART) has converted human immunodeficiency virus (HIV) from a once fatal diagnosis to a manageable chronic condition [1], resulting in a growing demographic of older adults living with HIV (OLHIV). These older adults now face the dual challenges of managing their condition while navigating the complexities of aging [1]. Globally, there are conceptual issues around the age classification of older adults living with HIV/AIDS (human immunodeficiency virus/acquired immunodeficiency syndrome) and are above 50 years, with an estimated 6 million people worldwide [2]. In this study, the term older adults living with HIV/AIDS refers to individuals aged 50 years and above who have been diagnosed with HIV or AIDS. While the global definition of older adulthood traditionally begins at age 60 or 65, research on HIV/AIDS commonly adopts the lower threshold of 50 years due to accelerated physiological aging associated with the disease, prolonged exposure to antiretroviral therapy, and increased susceptibility to comorbidities [3]. HIV is a virus that attacks the immune system and can lead to immune deficiency over time. AIDS is the most advanced stage of HIV infection, characterized by a severely weakened immune system and the presence of specific opportunistic infections or cancers [4]. Thus, recognizing this age group as “older adults” provides clarity in capturing their unique healthcare needs, social experiences, and vulnerabilities, particularly within the context of the COVID-19 pandemic. Of this population, about 3 million reside in the sub-Saharan regions of Africa [5], with about 13% living in Nigeria [6]. Unfortunately, the absence of an operational national aging policy or safety net services and programs [7] poses a unique challenge to the increasing population of Nigerians aging with HIV and their families.

The COVID-19 pandemic exposed and intensified existing inequities within public health systems and social structures, revealing the fragility of global health governance and the uneven distribution of care resources. Structurally marginalized populations—including racialized communities, the older adults, migrants, and low-income groups—bore the brunt of both the virus and the policy responses to it [8,9]. The pandemic illuminated how social determinants of health—such as housing, income, and access to healthcare—are not peripheral but central to health outcomes [10-12]. Psychosocially, the pandemic fueled widespread mental health crises, driven by isolation, grief, economic precarity, and uncertainty [12]. Lockdowns, while necessary for infection control, also exacerbated domestic violence, disrupted education, and intensified loneliness, especially among older adults and caregivers [13].

The intersection of aging and HIV in low-resource settings like Nigeria amplifies a syndemic of health vulnerabilities, social isolation, and economic hardship. Aging’s natural physiological decline, compounded by the chronic inflammation associated with HIV [14], accelerates age-related diseases [14,15] and increases morbidity and mortality [16]. This synergistic effect results in multimorbidity, ageism, stigma, social isolation, and diminished quality of life [17]. Social stigma from HIV further amplifies these difficulties, fostering isolation, loneliness, and anxiety [18]. Financial challenges, driven by disability, unemployment, inadequate retirement savings, and a lack of aging policy and adequate social welfare for older adults, further compound health vulnerabilities and economic hardship.

While COVID-19 challenged healthcare systems worldwide and disrupted routine care [19], it exacerbated the vulnerabilities of those living with chronic illnesses. For OLHIV, the pandemic introduced additional layers to existing challenges of co-occurrence of aging and HIV infection [19-21], including severe COVID-19 outcomes due to compromised immune systems, interruptions in HIV treatment and care due to healthcare system overload [22,23], and increased social isolation [24] resulting from public health measures. Furthermore, older Nigerians with HIV have to strive against the existing backdrop of extreme poverty [17], the stigma associated with having HIV [25], socioeconomic conditions, e.g., infrastructural inadequacies and economic instability [26], and the lack of specific resources in the care and support of older adults [27].

While there is a growing body of literature globally highlighting the challenging experiences of older adults living with HIV/AIDS during the pandemic, these studies have focused on the decline in mental health marked by depression [28], anxiety [29], stress [30], and fear [31]. Others highlighted the financial challenges [31], difficulties in accessing healthcare [24,31], and adherence to antiretroviral therapy (ART) and medication [24]. Very few studies have focused on the lived experiences of older adults living with HIV/AIDS during the pandemic within the African context.

This study adopts a social constructivist paradigm, which views realities as socially constructed through human interactions [32]. It prioritizes understanding lived experiences from the perspectives of those directly impacted, offering a lens to explore how older adults with HIV navigated the dual challenges of managing chronic illness during the COVID-19 pandemic [33]. This approach is particularly suited for narrative methods where individuals’ stories and the description to the stories they attach to their experiences are central. The constructivist lens provides insights to how older adults negotiate overlapping challenges of aging, chronic illness, and external crises, while adapting to disruptions in healthcare and social systems. This exploration can also offer insights into their resilience, coping mechanisms, and the socioeconomic and psychological impacts of the pandemic on this vulnerable population. The COVID-19 pandemic’s disruption of healthcare services underscores the need to explore the lived experiences of older adults living with HIV in resource-limited settings to improve healthcare systems that can address their specific needs, particularly during crises [34,35].

Uniquely, the overarching objective of this study is to explore the factors that shape experiences of older adults living with HIV/AIDS in Nigeria during the COVID-19 pandemic. This study fills an important gap by critically foregrounding the lived experiences of older adults living with HIV/AIDS during the COVID-19 pandemic, an often-overlooked demographic in public health discourses. While global and national HIV responses have prioritized younger populations, older adults face unique intersections of ageism, stigma, comorbidities, and systemic neglect. Therefore, centering their narratives within the Nigerian context, this research challenges age-blind health policies and exposes the structural inequities that shape access to care. It contributes to a more inclusive understanding of HIV and pandemic vulnerabilities, advocating for responsive, age-sensitive, and equitable health interventions in both policy and practice.

## 2. Literature Review on Older Adults Living with HIV/AIDS

Nigeria presents a critical case study within the African context, as it harbors the second largest HIV epidemic globally. As of 2018, about 13% of Nigerians living with HIV were aged 50 or above—roughly 280,000 older individuals. This cohort has grown, as ART scale-up enables long-term survival, yet older PLHIV have not been a traditional priority in Nigeria’s national HIV response [36]. Many have lived with the virus for years and now contend with age-related illnesses and socioeconomic challenges. A national geriatric HIV cohort study in Nigeria documented a high prevalence of chronic comorbidities in this group: for example, 21% had hypertension alongside HIV [37]. Such comorbid conditions heighten the vulnerability of older Nigerian PLHIV to infections like COVID-19. When the COVID-19 pandemic hit, Nigeria imposed lockdowns and mobility restrictions that significantly impacted healthcare access. Routine HIV services were disrupted, as clinics reduced hours and patients feared exposure to the coronavirus. A study from Lagos reported that people with HIV faced myriad difficulties during the lockdown, with psychological distress (anxiety and fear) reported by 78.5% of patients and widespread financial and food insecurity also noted [38]. These stressors were likely even more pronounced for older patients, who are at higher risk of severe COVID-19 and may have been more isolated. Many older PLHIV avoided hospitals due to fear of contracting COVID-19, echoing the sentiment *“I was afraid to go to the hospital”* observed among older patients in similar African settings. Nonetheless, some treatment interruptions occurred, and maintaining viral suppression in this period was challenging. Beyond medical care, support networks for older PLHIV in Nigeria were tested during the pandemic. Traditionally, family members (especially in multigenerational households) and community support groups provide care and reduce loneliness for older adults with HIV. COVID-19 lockdowns, however, limited in-person contact, potentially exacerbating feelings of loneliness and depression in a cohort already prone to mental health struggles [39]. Moreover, the stigma surrounding both HIV and COVID-19 may have further marginalized older individuals, deterring them from seeking help. Notably, Nigeria’s HIV epidemic intersects with other public health challenges, like tuberculosis (TB)—the country has one of the highest TB burdens globally, and HIV/TB co-infection is an increasing concern (https://businessday.ng/editorial/article/curbing-hiv-aids-scourge-amidst-covid-19-pandemic/#:~:text=Curbing%20HIV/AIDS%20scourge%20amidst%20Covid,an%20increasing%20concern%20for (accessed on 25 May 2022)). An older person living with HIV and latent TB, for instance, faces compounded risks if infected with COVID-19, which can severely affect the lungs. These intersecting comorbidities underscore the need for integrated care approaches. Despite the adversities, older PLHIV in Nigeria have demonstrated resilience. Many long-term survivors drew upon coping mechanisms and prior experiences of overcoming illness stigma. As one positive outcome, the pandemic spurred greater recognition by Nigerian health policymakers that older adults with HIV require targeted support. Efforts are now geared toward incorporating geriatric care principles into HIV services—for example, by monitoring age-specific treatment outcomes and strengthening community-based support for older patients. Going forward, lessons from COVID-19 are informing more inclusive healthcare strategies, ensuring that older adults living with HIV are not left behind in both pandemic responses and the broader HIV/AIDS care.

As PLHIV live longer, they face unique health challenges. Long-term HIV infection and prolonged ART use contribute to accelerated aging and a higher burden of comorbidities, such as cardiovascular disease and diabetes. Studies have noted that older HIV patients often manage multiple conditions simultaneously (polypharmacy) and have elevated risks of morbidity and mortality compared to younger adults [29,31,39]. Psychosocial concerns are also prominent: nearly 40% of older PLHIV exhibit symptoms of depression, and many report high levels of loneliness and social isolation [39]. HIV-related stigma intersects with ageism, limiting social support from family and community networks for older individuals. These pre-existing vulnerabilities were magnified during the COVID-19 pandemic. Older adults—especially those with HIV and underlying conditions—were at increased risk of severe COVID-19 outcomes, while pandemic restrictions disrupted routine HIV care. In many settings, HIV testing and clinic visits were delayed or paused as healthcare systems shifted focus to COVID-19 [28,38,39]. Telehealth emerged as a partial solution in high-income contexts, yet some older patients struggled with technology or preferred in-person care (https://pmc.ncbi.nlm.nih.gov/articles/PMC7755731/#:~:text=Author%20SBW%20is%20Chief%20of,needs%20of%20the%20older%20population (accessed on 25 May 2022)). Thus, the convergence of HIV and COVID-19 created a syndemic scenario wherein health access, treatment adherence, and mental wellbeing of older PLHIV were put under additional strain. Across global, African, and Nigerian contexts, the COVID-19 pandemic highlighted and intensified the multidimensional challenges faced by older adults living with HIV/AIDS. Key themes emergent in the literature include healthcare access barriers during lockdowns, heightened psychosocial distress, persistent stigma, a high burden of comorbidities, and the crucial role of support systems in sustaining care and wellbeing. Academic studies and reports by agencies such as the WHO and UNAIDS consistently call for differentiated interventions for this demographic [34,40]. Addressing the needs of older PLHIV—from integrating HIV and NCD care to combatting stigma and bolstering social support—is essential for an effective HIV response, especially in the wake of COVID-19. The pandemic has ultimately prompted a re-examination of how health systems can better protect vulnerable aging populations. Therefore, learning from these experiences, stakeholders can craft more resilient health services and community support networks that ensure older people living with HIV in Nigeria, Africa, and worldwide receive the comprehensive care and respect they deserve, even amid future public health crises.

## 3. Methodology

### 3.1. Study Design

This study employed a narrative inquiry approach to explore the lived experiences of older adults (aged 50 and above) living with HIV during the COVID-19 lockdown at a tertiary HIV care clinic in Ibadan, Nigeria. Narrative inquiry was deemed methodologically appropriate for its capacity to illuminate the storied nature of human experience—moving beyond individual accounts to reveal how personal, social, and structural contexts shape meaning-making [41]. Rooted in the belief that individuals make sense of their lives through storytelling, this approach enabled a nuanced examination of how participants constructed, interpreted, and gave coherence to their experiences amid overlapping crises. In-depth interviews served as the primary mode of data collection, allowing participants to narrate their experiences in their own words and on their own terms. Through these rich, contextually embedded narratives, the study sought to identify emergent patterns, and to surface the complex emotional, relational, and institutional dimensions of living with HIV in later life during a global pandemic [42].

### 3.2. Study Setting

The study was conducted at the HIV clinic of the Infectious Diseases Institute (IDI) of the College of Medicine, University of Ibadan (IDI/CoMUI), located in Ibadan, the capital of Oyo State in southwestern Nigeria. As Nigeria’s third most populous city and a regional hub for health services, Ibadan presents a critical site for examining the intersections of urban public health infrastructure and long-term HIV care. As of mid-2025, its metropolitan population is projected to be approximately 4.14 million, a 3.5% growth from the previous year—while its urban agglomeration is estimated at around 3.65 million [43]. The IDI clinic is one of Nigeria’s pioneering HIV treatment centers, established in 2002 as part of the national scale-up of antiretroviral therapy (ART). Since 2004, it has received significant support from the U.S. President’s Emergency Plan for AIDS Relief (PEPFAR), facilitating the expansion of services and enhancing access to comprehensive HIV care [44]. The clinic operates a structured care system, where people living with HIV (PLHIV) receive monthly appointments for medication refills and clinical consultations. As of November 2022, 2851 older adults (aged 50 and above) were actively receiving ART at the IDI clinic—a reflection of the increasing longevity of PLHIV in Nigeria and the growing need for agesensitive HIV services. The choice of this site was therefore both strategic and appropriate. It enabled access to a large, diverse, and stable population of older adults managing HIV in a context where ART has been consistently delivered over two decades. Moreover, the clinic’s institutional history, combined with its centrality in Nigeria’s HIV response, provided a robust backdrop for exploring how structural, relational, and temporal factors shaped older adults’ lived experiences during the COVID-19 lockdown. From a narrative inquiry perspective, the setting offered a rich context for eliciting storied accounts of resilience, disruption, and continuity in care across overlapping health crises.

### 3.3. Participants and Procedures

A purposive sampling strategy, guided by maximum variation principles, was employed to ensure a diverse range of perspectives among older adults living with HIV. Key variation criteria included gender, age, and educational attainment, which are known to influence health-seeking behaviors and care experiences. Inclusion criteria required that participants be aged 50 years or older, have been on antiretroviral therapy (ART) for at least two years prior to the onset of the COVID-19 lockdown, and be willing to provide informed consent, including permission for audio-recording of interviews. Individuals who did not meet the age threshold, had initiated ART less than two years before the lockdown, or were unable to participate due to cognitive or health-related constraints were excluded from the study. Recruitment occurred in-clinic and continued until thematic saturation was reached, ensuring depth and breadth of insight. Notably, variation in the duration of HIV infection ranged from those diagnosed in the early 2000s to more recent cases, which yielded important differences in participants’ narratives. Long-term survivors often framed their experiences through the lens of historical resilience, having previously navigated stigma, treatment access barriers, and health system transformations. In contrast, participants with more recent diagnoses emphasized uncertainty, heightened anxiety about dual risk (HIV and COVID-19), and reliance on evolving digital or community-based support systems. This variation enriched the data, enabling a nuanced understanding of how the temporality of illness shaped older adults’ experiences of care, risk perception, and adaptive strategies during the pandemic.

An interview guide was developed for this study by the primary investigator, divided into four sections: *Section A* inquired about the participants’ concerns and perspectives about the COVID-19 pandemic, *Section B* explored the impact of the lockdown on their wellbeing as well as their physical, psychological/mental, and social wellbeing, *Section C* inquired about the effect of the lockdown on their treatment at the IDI, while *Section D* inquired about how they navigated the challenges described and the coping strategies they engaged. Two interviewers piloted the guide with four initial participants before administering the guide to a larger sample. Using an iterative process, face-to-face key informant interviews were conducted in two rounds, with the interview guide refined after the initial round (first eight interviews) to include probes on access to specific services (e.g., bleeding and counseling) during the lockdown. All interviews were conducted face-to-face with the participants, audio recorded, and transcribed for analysis.

### 3.4. Data Analysis

We employed a narrative analysis approach to explore the meaning-making processes embedded in participants’ accounts, with particular attention to the ways in which older adults constructed and interpreted their experiences of living with HIV during the COVID-19 lockdown. Narrative analysis was chosen for its emphasis on stories as socially situated texts that reflect broader contexts, temporalities, and identities. The analytic process was iterative, reflective, and inductively grounded in the data. Authors O.O.E. and O.O. led the three-stage analytic process. First, each transcript was read multiple times to develop a holistic understanding of participants’ narratives, allowing for immersion in the content and context. In the second stage, we organized the data inductively, using open coding to identify significant phrases, experiences, and patterns of meaning. Codes were initially descriptive and gradually developed into more interpretive and analytical categories. Through constant comparison, similar codes were grouped and refined into a hierarchical structure comprising grandparent, parent, and child coding nodes.

NVivo 12 software was employed to facilitate data management and analytical organization. Both analysts (O.O.E. and O.O.), proficient in the use of NVivo, constructed coding trees that reflected layered thematic relationships. The process emphasized participant meaning over semantic expression, focusing on what was said rather than how it was said. An initial codebook, including the emerging coding schema, was circulated among all authors for collaborative reflection and refinement. Themes were then constructed by tracing connections across narratives, identifying recurring patterns and variations in participants’ storied experiences. Differences in thematic interpretation were resolved through group discussion, revisiting transcripts and interview memos to ensure coherence and rigor.

Importantly, the diverse duration of HIV diagnosis among participants—ranging from long-term survivors to more recently diagnosed individuals—surfaced distinct narrative patterns. Long-term survivors often framed their experiences through resilience and continuity, whereas more recent diagnoses emphasized disruption, fear, and uncertainty. These narrative contrasts were central to theme development and interpretation.

To enhance trustworthiness, member checking was conducted with eight participants, who reviewed selected emerging codes and themes for accuracy and resonance. Their feedback informed the final thematic structure and ensured that the findings remained grounded in the participants’ lived realities. Overall, the collaborative and reflexive nature of the analysis aimed to preserve narrative integrity while generating a rich, contextually embedded understanding of older adults’ experiences of HIV care during the pandemic.

## 4. Results

A total of 26 participants (13 males and 13 females) were enrolled and interviewed for this study between March and April 2023. The participants’ ages ranged between 51 and 79 years of age, with the majority aged 51–59 (15 of 26) and married (12 of 26). Educational backgrounds varied, with 2 participants having had no formal education and the plurality having a tertiary-level of education (12 of 26). The participants’ occupations varied, with the majority self-employed (18 of 26), 4 were civil servants, and 4 participants mentioned they were retired and unemployed. The number of years living with HIV ranged from 5 to 21 years (Table 1).

### 4.1. Overview of Findings

The narratives revealed five interrelated themes that illuminated the complex experiences of older adults living with HIV during the COVID-19 lockdown. Financial concerns emerged prominently, with most participants describing heightened food insecurity and economic strain due to disrupted livelihoods. These hardships were closely tied to their health, as the inability to maintain proper nutrition compromised adherence to antiretroviral therapy (ART), illustrating a clear intersection between economic survival and medical compliance. While some found stability through continued employment or family support, many faced trade-offs between food intake and medication adherence. Access to treatment was also disrupted, with accounts reflecting both increased stress due to overcrowded clinics and relief from revised appointment scheduling that reduced visit frequency. The psychological impact of the lockdown ranged from anxiety and depression that were rooted in fear of COVID-19 and HIV stigma, to moments of emotional connection and familial bonding. Coping strategies varied widely, from drawing on religious faith and resilience to engaging in recreational activities and relying on social networks for sustenance and care. Participants’ stories underscored the uneven terrain of living with HIV during a public health crisis. While some experienced relief and stability, others faced compounding vulnerabilities (Table 2).

### 4.2. Theme 1: Financial Concerns

The COVID-19 lockdown significantly exacerbated financial constraints among older adults with HIV/AIDS, impacting their ability to afford and access sufficient food. This was primarily due to the closure of businesses, leading to reduced income or complete loss of earnings for many. Moreover, some were widowed, single earners without partners to support financially during this period when personal businesses and trading were shut down. The direct consequence of this financial strain was a notable deterioration in nutritional wellbeing, as participants struggled to purchase food, forcing them to compromise on the quality and quantity of their meals. Participants expressed a significant intricate link between financial stability and the ability to maintain a healthy diet, particularly for those with chronic health conditions. A participant indicated that:

“My major concern during COVID-19 lockdown was that I had difficulty feeding; there was no means of making and getting money for feeding. . .”

(PT15., 57 years old)

The participants were asked about the financial constraints and particular needs that were financially hampered during the pandemic. A 69-year-old patient mentioned that:

“The impact concerned the rule to keep a certain distance from each other. I could not feed well; I only fed on whatever food was available. Even though I had been restricted from taking certain food like garri, I could not abide by it due to the lockdown.”

(PT1., 69 years old)

In contrast, a positive experience from a respondent stated that they were getting paid by their employing company even while the compulsory stay-at-home regulation was ongoing:

“The impact of the lockdown was fortunate for me because even during the lockdown, my company was still paying me. So economically, to be honest, it was not something I felt so badly.”

(PT4., 50 years old)

### 4.3. Theme 2: Adherence to Antiretroviral Therapy

The financial strain caused by the lockdown profoundly affected individuals living with HIV, creating a ripple effect that undermined their ability to maintain both proper nutrition and consistent adherence to antiretroviral therapy (ART). For many, the lockdown meant not only a loss of income but also limited access to basic necessities, leaving them grappling with the challenge of securing sufficient food to sustain their health. One respondent shared her poignant experience, recounting how the scarcity of food made it difficult to adhere to her ART regimen as prescribed: *“I couldn’t take my medication properly,”* she explained, *“because I wasn’t eating regularly.”* Her words reflect the harsh reality faced by many: ART, though a lifesaving treatment, often requires regular meals to minimize side effects and ensure effectiveness. The lack of food not only exacerbated physical challenges but also added to the emotional toll, as individuals were forced to choose between enduring the medication’s side effects on an empty stomach or skipping doses altogether:

“It was a necessity to me that I must take my medication as and when due. However, things didn’t go so well at that time, as we had to eat before taking drugs and if we didn’t eat we couldn’t take the drugs; it’s not right to take drugs on empty stomachs.”

(PT11., 68 years old)

For some individuals, the lockdown unexpectedly brought a silver lining: the chance to consistently adhere to their antiretroviral therapy (ART) regimen. The enforced time at home removed the distractions and allowed them to establish a regular schedule for medication intake. One respondent noted that staying at home made it easier to remember their medication. Additionally, the heightened health education campaigns during this period had a profound impact. Tailored to raise awareness about staying safe, these initiatives provided clearer guidance on the proper use of ART, including understanding dosages and the therapy’s protective benefits against opportunistic infections:

“I never knew the medication I take has a certain time of consumption but I became aware of it during the lockdown. . .”

(PT1., 69 years old)

### 4.4. Theme 3: Impact on Access to Treatment

The experiences of participants in accessing treatment during the lockdown revealed a spectrum of challenges, underscoring the profound impact of the pandemic on healthcare systems. For many, the lockdown measures significantly disrupted the routine operations of clinics, increasing waiting times and transforming once predictable schedules into sources of uncertainty:

“It was bad, it really affected us as there were a lot of people on the queue when we came for our drugs. We mostly had to wait or either go back and come back later.”

(PT11., 68 years old)

Conversely, the introduction of extended appointment schedules, from the usual three-monthly to six-monthly appointments, was a welcome change for others:

“The six-month appointment started at that time, and it’s even better for us, too. We do not enjoy coming here.”

(PT4., 50 years old)

This adjustment alleviated the burden of frequent clinic visits, highlighting an adaptive response to healthcare delivery during the pandemic that could have lasting benefits.

### 4.5. Theme 4: Psychological Impact of COVID-19 Lockdown

The psychological impact of the COVID-19 lockdown on patients attending the IDI clinic in Ibadan painted a complex and deeply personal spectrum of experiences. For many, the pandemic brought overwhelming emotions, such as fear, anxiety, depression, and a profound sense of loneliness or isolation. These feelings were often exacerbated by the uncertainty surrounding the virus, the disruption of routine care, and the stigma tied to living with HIV/AIDS. Yet, amid these challenges, some patients described unexpected moments of satisfaction and strengthened family bonds. The forced slowdown of daily life allowed for deeper connections within households, providing comfort and emotional support during a time of crisis. This stark bifurcation in experiences reveals the multifaceted psychological toll of the pandemic, particularly on individuals with pre-existing health conditions.

### 4.6. Fear, Anxiety, and Depression

For some individuals, the lockdown period was a time of overwhelming fear, anxiety, and depression, as the pandemic introduced new uncertainties. Participants experienced fears of heightened susceptibility to COVID-19, creating an intense psychological distress. Their words encapsulate the emotional turmoil faced by many during this time:

“At the first stage of the outbreak, there was no cure for it, and that caused much death, so that put fear in me.”

(PT19., 79 years old)

“I was anxious because I used to come here. When I reached home, the clothes that I wore, I was even afraid of taking the clothes inside because of my children; I was afraid, I was afraid to let them come on me to get it too. . .”

(PT8., 51 years old)

“Anxiety, it is because of my status. Because I have HIV, I pray do not let me have COVID-19 again. . . even depression, I had depression because of my status.”

(PT17., 56 years old)

“. . .I was sad due to financial constraints. I was always scared.”

(PT1., 69 years old)

Another shared a similar concern about the risk of contracting COVID-19, given their health status:

“My concern was how we, judging by this our status, can get COVID-19 easily. So that was my major concern during COVID-19. It was only my status.”

(PT17., 56 years old)

The inability to engage in social activities further exacerbated feelings of loneliness, as one respondent lamented:

“We couldn’t move out, it affected me. . . . I wasn’t really fulfilled at all; it really affected my social life.”

(PT4., 50 years old)

### 4.7. Satisfaction and Bonding

In contrast to the widespread challenges, some participants found unexpected solace in the lockdown experience. For them, the enforced isolation brought about positive psychological effects, offering a chance to slow down and reflect. One respondent shared how the lockdown became an opportunity for personal growth and deeper familial connections. For these individuals, the stillness of the lockdown allowed space for introspection and meaningful relationships, transforming an otherwise challenging period into an opportunity:

“Well, back then, it played a good role for me... It made people realize that nothing in this world is worth good health or good wellbeing, which is very important, but I can gracefully say, it was an advantage.”

(PT11., 68 years old)

This sentiment was echoed by others who found joy in simple activities and the company of family members:

“I am someone that loves seeing movies, so whenever there was light, I spent the most time watching movies.”

(PT14., 51 years old)

“My children and husband were there for me to relate with, so I did not feel the impact; it was even as if the lockdown should continue because I was just enjoying myself with my family since we love each other.”

(PT14., 51 years old)

These reflections underscore individuals’ adaptability to finding satisfaction and fostering stronger family bonds despite the challenges of the lockdown.

### 4.8. Theme 5: Coping with or Navigating Challenges

The lockdown during the COVID-19 pandemic brought a myriad of challenges, yet it also underscored the critical role of social and institutional support systems embedded within many Nigerian communities. Amid the economic and emotional strain, these networks became a lifeline for individuals navigating the uncertainties of the crisis. For some participants, financial and social support from family, friends, and religious organizations served as a crucial buffer against the lockdown’s harsh impacts. One respondent shared how relatives and faith-based groups stepped in to provide food and financial assistance, helping to alleviate the burden of lost income:

“I received some things from my church to help financially; that was where I received help from.”

(PT5., 76 years old)

“My family had been so supportive. . .not only financially, they constantly reached out to know my wellbeing.”

(PT13., 66 years old)

“I made myself happy by spending time with my family, interacting with some of my colleagues.”

(PT17., 56 years old)

Others leveraged their resilient attitude, religious inclination, and participation in recreational activities as a means of coping with the adverse effects of the lockdown. For instance:

“Well, I understood that all those measures were just for a while, so I learned to endure it since they were all geared towards our wellbeing.”

(PT24., 65 years old)

“Endurance and long-suffering, that’s what we needed to overcome those days...It’s all about determination.”

(PT11., 68 years old)

### 4.9. Religiosity

“. . .I related it to a friend, and she told me to put my trust in God, and that was exactly what I did, so the sadness went away.”

(PT23., 59 years old)

### 4.10. Participating in Recreational Activities

“. . .there is a field by my house...if I see where they are playing ball, I go there... These are the things that keep me on, that keep me happy. I was playing table tennis during the lockdown. I have table tennis at home.”

(PT5 76 years old)

Although, some participants mentioned receiving reminder calls about their appointments and drug pickup. However, there was no mention/reference to the availability of psychological or mental health support for the participants during the lockdown. These varying levels of support and their impacts on individuals’ experiences highlight the heterogeneity within the population and underscore the need for targeted interventions to address the specific needs of older adults with HIV/AIDS during crises.

## 5. Discussion

The COVID-19 lockdown significantly exacerbated financial instability among older adults living with HIV/AIDS (OLHIV) in Nigeria, with direct implications for antiretroviral therapy (ART) adherence and nutritional wellbeing. Our study revealed that dependent on informal, daily income-generating activities, experienced abrupt income loss due to business closures. This financial strain undermined their ability to maintain treatment routines and proper nutrition, which are both essential for ART efficacy, echoing similar evidence from Uganda showed reinforced need for crisis-responsive social protection mechanisms [45]. This financial strain compounded the challenges of ART adherence, as several participants reported skipping doses or delaying refills due to the inability to afford the nutrition needed for safe medication intake [29]. This signals a critical, yet often overlooked, connection between economic stability and chronic disease management, where inadequate nutrition further weakens immune function and amplifies ART side effects, creating a negative feedback loop that compromises long-term health outcomes [46,47]. The absence of targeted financial support, as seen in southern African contexts where governments provided direct cash transfers and home-based care [24], further deepened this crisis in Nigeria, exposing significant policy gaps that undermine the health and resilience of OLHIV.

The pandemic also exposed systemic weaknesses in Nigeria’s healthcare infrastructure, particularly its limited capacity to provide integrated, age-sensitive care. Older adults with HIV reported significant disruptions in accessing routine services for comorbidities, such as hypertension and diabetes, conditions that require regular monitoring. These challenges, compounded by fear of infection and overwhelmed facilities, reflect broader global trends in chronic disease management during health crises and underscore the urgent need for resilient, person-centered, and pandemic-responsive healthcare systems [34,48]. This neglect heightened the risk of unmanaged comorbidities, exacerbating the physiological vulnerabilities associated with aging with HIV, such as immune dysregulation, cardiovascular complications, and metabolic disorders [15]. The resulting decline in quality of life underscores how health system gaps disproportionately affect older adults living with HIV (OLHIV). However, the introduction of extended medication refill intervals and limited telehealth services during the lockdown was viewed positively by some participants, indicating that these adaptive strategies may offer a sustainable pathway toward more flexible, patient-centered care delivery post-pandemic [49]. Such interventions not only reduce clinic congestion and the risk of infectious exposure but also address mobility and financial barriers commonly faced by older adults. These findings highlight an urgent need for the implementation of innovative, integrated care models that holistically address both HIV and age-related conditions. Specifically, scaling up differentiated service delivery (DSD) models—such as multi-month dispensing, decentralized drug pick-up points, and targeted teleconsultations—could enhance treatment continuity, reduce systemic strain, and improve health outcomes for OLHIV. Importantly, these models must be designed with attention to digital literacy, social support, and equity to ensure accessibility across socioeconomic and educational divides.

Psychological distress emerged as a critical theme, with participants describing profound fear, anxiety, and social isolation during the lockdown period. These emotional responses were fueled by both the uncertainty of the COVID-19 pandemic and the heightened perceived vulnerability associated with living with HIV, a pattern consistent with global evidence indicating that older adults with chronic conditions experienced significantly elevated mental health burdens during the pandemic [50]. For many, the fear of dual infection and the disruption of routine care exacerbated feelings of helplessness and loss of control. Yet, in contrast to this distress, several participants articulated a surprising sense of solace during the enforced stillness of the lockdown. This period provided space for self-reflection, reconnection with family, and renewed spiritual engagement. Such adaptive coping, often grounded in familial bonds and religious faith, underscores the complex interplay between vulnerability and resilience. These findings highlight an important but underexplored resource: the culturally embedded support systems that sustain emotional wellbeing among older adults living with HIV (OLHIV). From a practical perspective, mental health interventions for this population must move beyond individual pathology models to incorporate community-based, faith-informed, and intergenerational strategies. Tailoring psychosocial support to reflect these lived realities—such as integrating family education, peer mentorship, and spiritual counseling—can foster culturally congruent and sustainable pathways to emotional resilience [51]. Our findings underscore the crucial role of informal support networks in buffering older adults living with HIV (OLHIV) against the worst impacts of the COVID-19 pandemic. Participants frequently attributed their resilience and ability to sustain ART adherence to the financial, emotional, and instrumental assistance provided by family members and religious communities. This reliance on informal systems mirrors findings from other resource-limited contexts, where social capital often fills gaps left by insufficient formal health and social welfare infrastructure [52]. Scientifically, this highlights the importance of recognizing informal care as a key determinant of health outcomes, particularly in crises when official systems falter. Practically, integrating these informal support structures into health policy frameworks could strengthen community resilience, enhance adherence strategies, and mitigate broader psychosocial distress among OLHIV. Policymakers must, therefore, develop culturally sensitive, community-based models that leverage existing familial and faith-based networks. Examples include community ART distribution programs, caregiver training, peer-support initiatives, and collaborations between health systems and religious organizations. Such integrated interventions have potential to reinforce informal safety nets, optimize health outcomes, and reduce vulnerability among older populations during current and future health crises.

## 6. Conclusions

This study illuminated the intersecting vulnerabilities experienced by older adults living with HIV during the COVID-19 lockdown in Nigeria. Financial instability emerged as a pervasive stressor, directly compromising nutritional health and treatment adherence, thus exacerbating physiological risks inherent in aging with HIV. While healthcare disruptions highlighted systemic inadequacies, they also catalyzed adaptive innovations, such as extended medication schedules and enhanced health communication. Psychological experiences were markedly heterogeneous—pervasive anxiety, depression, and isolation co-existed alongside resilience and emotional renewal fostered through strengthened familial bonds and reflective solitude. The pivotal role of informal support networks, such as family, community, and faith-based resources, demonstrated their critical, though often invisible, function in sustaining wellbeing amidst inadequate formal structures. These findings revealed the urgent necessity of holistic, culturally resonant interventions integrating informal care systems into formal health policy. Future strategies must prioritize structural resilience, embracing flexible healthcare models, psychosocial support that acknowledges community assets, and targeted economic safety nets, thus ensuring equitable and sustainable support for this marginalized population.

## 7. Limitations

The findings of this study must be considered in light of several limitations inherent in its qualitative narrative design. First, the purposive sampling from a single tertiary HIV care clinic in Ibadan, southwestern Nigeria, limits the transferability of the findings. Although maximum variation was sought to capture diverse perspectives, the narratives reflect the specific sociocultural and healthcare dynamics of this region and, therefore, experiences may differ substantially in other geographic or socioeconomic contexts. Additionally, data collection occurred retrospectively, several months following the peak of the COVID-19 lockdown, potentially influencing participants’ ability to recall precise details and emotions associated with their experiences. This temporal gap may have resulted in selective memory or retrospective reframing of experiences, introducing recall bias into the narratives. Further, the absence of real-time observations or complementary methods, such as diaries or longitudinal interviews, limited opportunities for deeper exploration of evolving experiences during the lockdown itself. Nevertheless, these limitations highlight opportunities for future research, advocating for multi-site studies, longitudinal approaches, and inclusion of real-time qualitative methods to enhance trustworthiness and depth in capturing the complexity of lived experiences.

## Figures and Tables

**Table 1.** Demographic characteristics of the participants.

Characteristics	Frequency	Percentage
**Age**		
50–59	15	57.7
60–69	7	26.9
70–79	4	15.4
**Gender**		
Male	13	50.0
Female	13	50.0
**Marital status**		
Married	12	46.2
Divorced	3	11.5
Widowed	11	42.3
**Educational Status**		
No formal education	2	7.7
Primary	5	19.2
Secondary	7	26.9
Tertiary	12	46.2
**Employment Status**		
Government employed	4	15.4
Self-employed	18	69.2
Unemployed	4	15.4

**Table 2.** Emerging themes, sub-themes, and a few representative quotes from the participants.

Theme	Sub-Theme	Quotes
Financial concerns	Employment stability	“The impact of the lockdown I would say was fortunate for me because even during the lockdown, my company was still paying me. So, economically, to be honest, it was not something I felt so badly.” (PT4., 50 years old)
	Difficulty in meeting basic needs	“My major concern during the COVID-19 lockdown was that I had difficulty feeding; there was no means of making and getting money for feeding.” (PT15., 57 years old)
Adherence to ART drugs	Dietary requirements	“It was a necessity to me that I must take my medication as and when due. However, things didn’t go so well at that time, as we had to eat before taking drugs and if we didn’t eat we couldn’t take the drugs; it’s not right to take drugs on empty stomachs.” (PT11., 68 years old)
	Awareness of medication regimen	“I never knew the medication I take has a certain time of consumption but I became aware of it during the lockdown.” (PT1., 69 years old)
Impact on access to treatment	Difficulty in seeking medical advice	“Sometimes, my BP would be high and I would like to see my doctor to ask her; ‘ma, can I change my drug’ and tell her the way I’m feeling but then, I was unable to do so, it’s been a long time since I last saw her. Ah, that affected us seriously.” (PT15., 57 years old)
	Changes in healthcare delivery	“I was coming to the hospital during that period but the doctors themselves reduced the frequency of appointments. They gave us precise dates at that time and once we came, we were given drugs that would last us for a while until our next appointment.” (PT17., 56 years old)
Psychological effects	Fear	“At the first stage of the outbreak, there was no cure for it and that caused a lot of death, so that put fear in me.” (PT19., 79 years old)
	Anxiety	“Anxiety, it’s because of my status. Because I have HIV, I pray don’t let me have COVID-19 too. ..” (PT17., 56 years old)
	Depression	“. . .even depression, I had depression because of my status.” (PT17., 56 years old)
	Satisfaction and bonding	“My children and husband were there for me to relate with, so I did not feel the impact, it was even as if the lockdown should continue because I was just enjoying myself with my family since we love each other.” (PT14., 51 years old)
	Loneliness and isolation	“We couldn’t move out, it affected me. ... I wasn’t really fulfilled at all; it really affected my social life.” (PT4., 50 years old)
Coping with or navigating challenges	Family support	“My family had been so supportive. . .not only financially they constantly reached out to know my wellbeing.” (PT2., 73 years old)
	Resilience	“Well, I understood that all those measures were just for a while so I learnt to endure it since they were all geared towards our wellbeing.” (PT24., 65 years old)
	Religiosity	“. . .I related it to a friend and she told me to put my trust in God and that was exactly what I did so the sadness went away.” (PT23., 59 years old)
	Participating in recreational activities	“. . .there is a field by my house...if I see where they are playing ball, I go there. . . These are the things that keep me on, that keep me happy. I was playing table tennis during the lockdown. I have table tennis at home.” (PT5., 76 years old)

## Data Availability

The data presented in this study are unavailable due to privacy concerns and ethical restrictions.
